# Plaque-Type Psoriasis Induced by Non-Steroidal Aromatase Inhibitor (Letrozole): A Case Series

**DOI:** 10.7759/cureus.107454

**Published:** 2026-04-21

**Authors:** Gaeda K Alkaltham, Amnah N Almulhim, Lenah Y Shaikh, Musaed Alsebayel

**Affiliations:** 1 Dermatology, King Fahad Specialist Hospital, Dammam, SAU

**Keywords:** breast carcinoma, letrozole, non-steroidal aromatase inhibitor, plaque-type psoriasis, psoriasis

## Abstract

Psoriasis is a multifactorial disease triggered by various factors, including medications. Letrozole is an effective adjuvant hormonal therapy used in the treatment of breast cancer and has been linked to different skin eruptions. However, psoriasis is not a well-established side effect. We describe two patients with similar manifestations of plaque psoriasis. Both cases were confirmed through histological examination. This case report highlights the importance of recognizing letrozole as a possible cause of plaque psoriasis.

## Introduction

Psoriasis is a chronic, immune-mediated inflammatory skin disorder characterized by erythematous, scaly plaques that commonly involve the elbows, knees, scalp, and trunk. It affects approximately 2-3% of the global population and arises from a combination of genetic predisposition and environmental triggers, including infections, stress, trauma, and certain medications. Beyond its cutaneous manifestations, psoriasis can significantly impair quality of life and is associated with systemic comorbidities such as psoriatic arthritis, cardiovascular disease, and metabolic syndrome [1].

Letrozole is a non-steroidal aromatase inhibitor widely used as adjuvant hormonal therapy in postmenopausal women with hormone receptor-positive breast cancer [2]. By reducing circulating estrogen levels, letrozole inhibits the growth of estrogen-dependent tumors [2]. While generally well tolerated, it has been associated with a range of cutaneous adverse effects, most commonly nonspecific rashes or pruritus [3]. Rarely, letrozole and other aromatase inhibitors may trigger inflammatory dermatoses, including psoriasis, likely due to estrogen depletion altering cutaneous immune regulation [3].

In this case series, we report two patients who developed new-onset psoriasis shortly after starting letrozole. We describe their clinical presentation, diagnostic evaluation, management, and outcomes, highlighting the importance of recognizing this uncommon but clinically significant adverse effect in patients receiving aromatase inhibitors.

## Case presentation

Case 1

A 69-year-old female with a history of hypertension (HTN) and relapsed breast cancer presented to the dermatology department in April 2024 with a skin eruption that began approximately 10 months earlier, six months after starting Letrozole (2.5 mg daily). She had no personal or family history of psoriasis or previous joint symptoms. She was recently diagnosed with ductal carcinoma in situ, which was estrogen- and progesterone-receptor positive, and underwent a right mastectomy.

She had been on amlodipine 10 mg for 30 years for hypertension. In 2011, she was diagnosed with T2N0 triple-negative breast cancer, underwent a right lumpectomy, and received four cycles of cyclophosphamide along with 35 radiotherapy sessions. In 2018, she underwent a total abdominal hysterectomy with bilateral salpingo-oophorectomy due to a diagnosis of adenomyosis.

**Figure 1:**
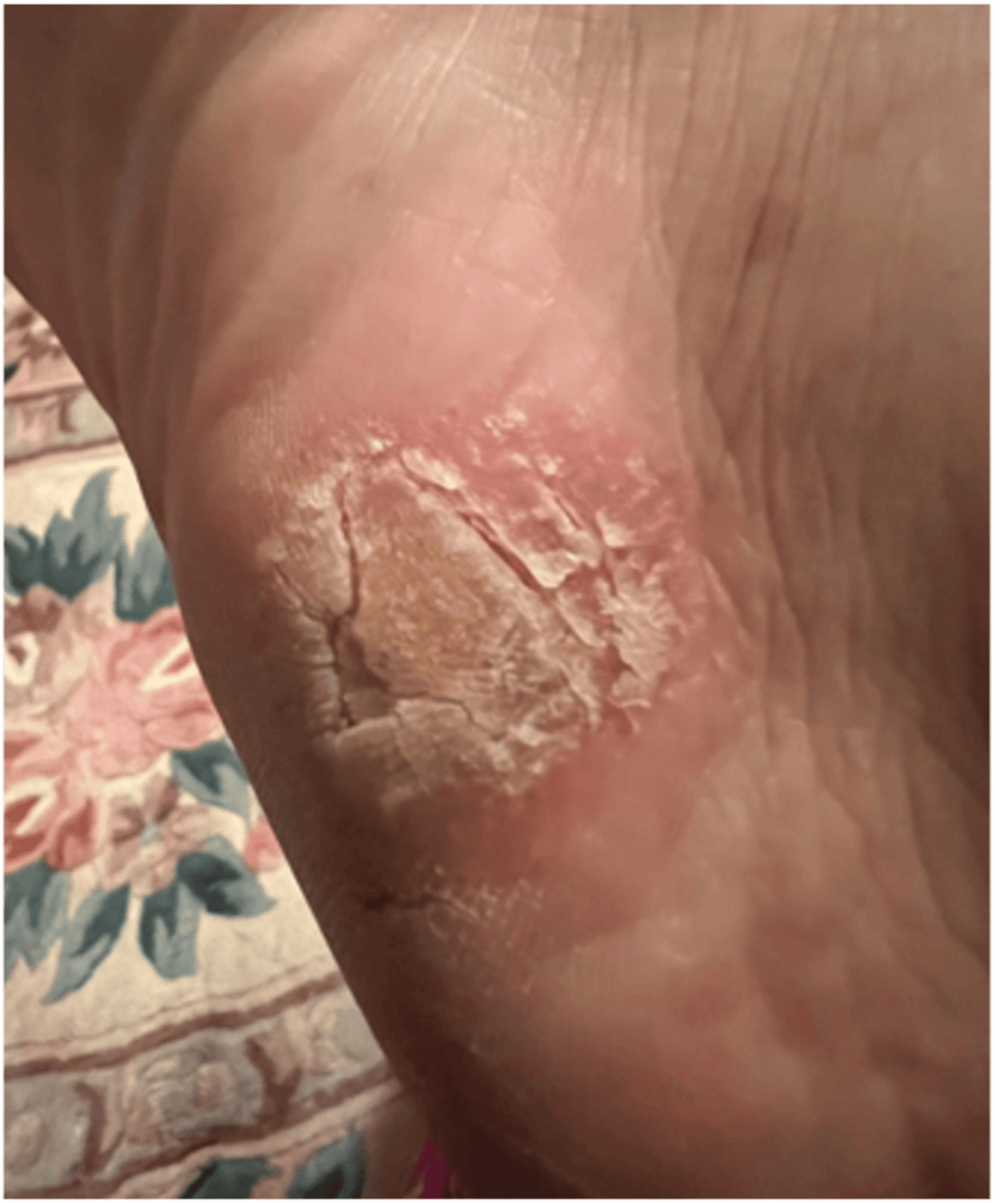
The plantar lateral aspect of the left foot

**Figure 2:**
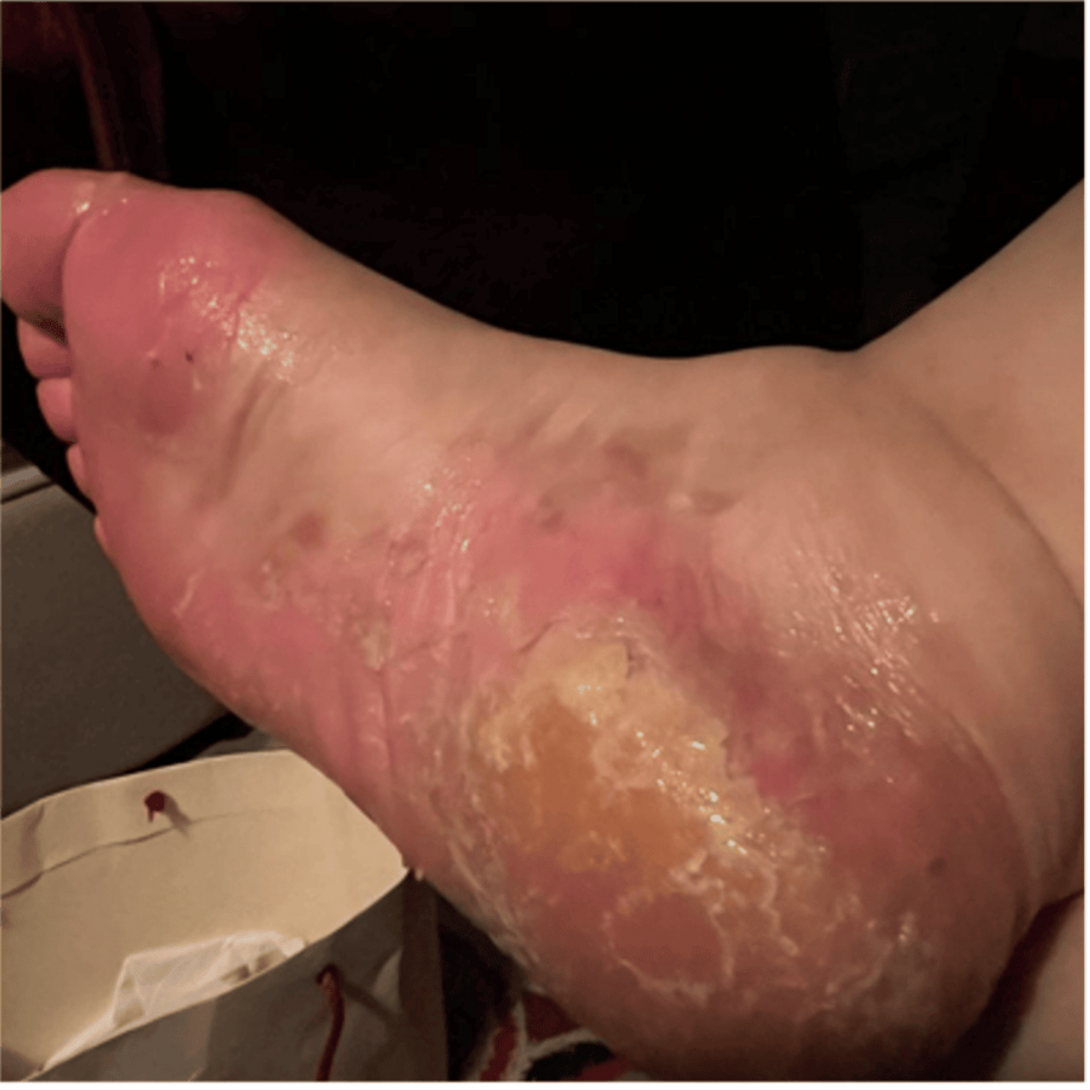
The plantar aspect of the right foot

**Figure 3:**
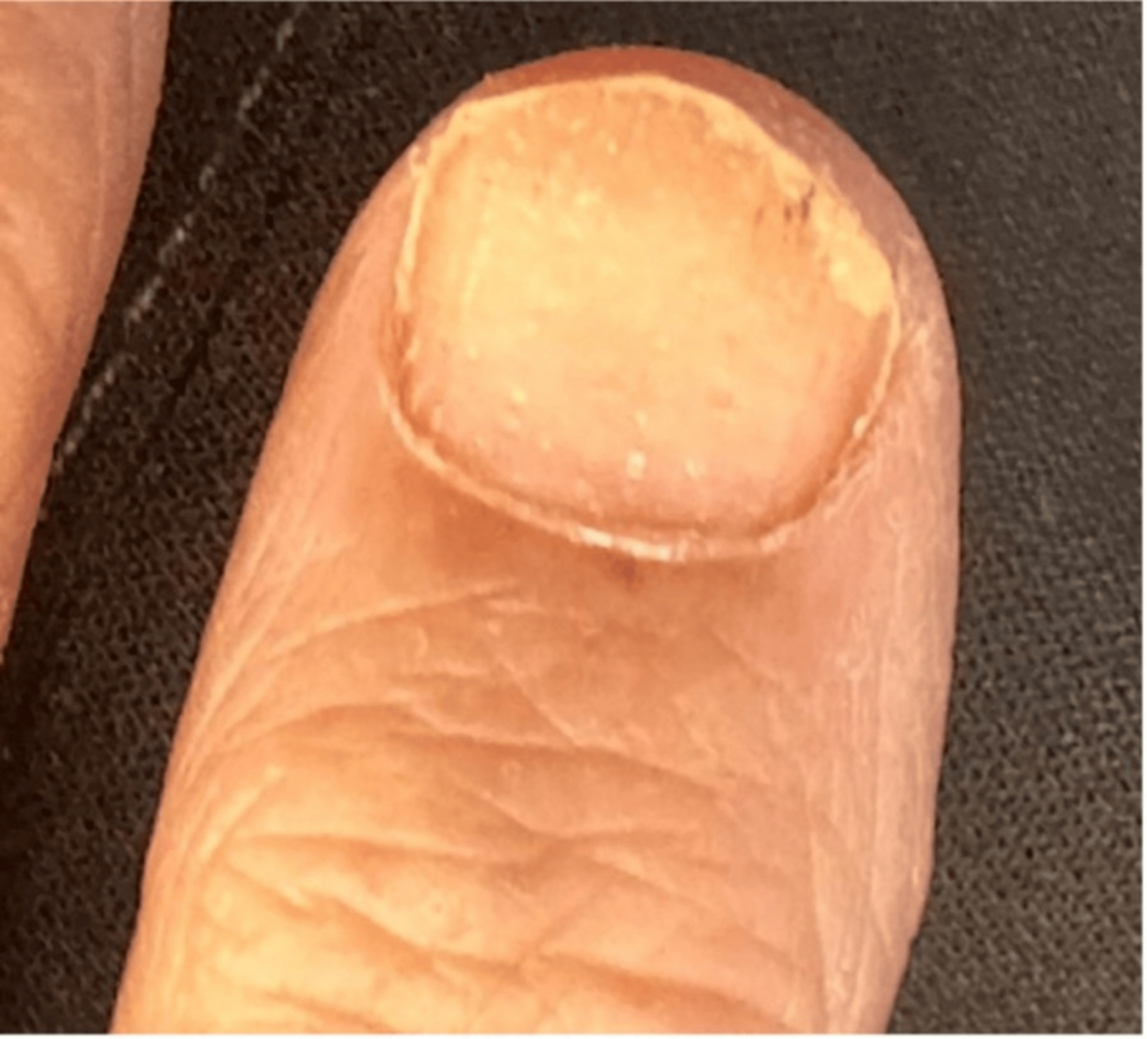
Nail changes of the index finger

On examination, she had bilateral, well-demarcated pink plaques with silvery scales over the palmoplantar surfaces (Figures 1, 2), elbows, and knees, along with nail changes, including pitting, oil spots, and onychorrhexis, involving all of her nails (Figure 3).

**Figure 4:**
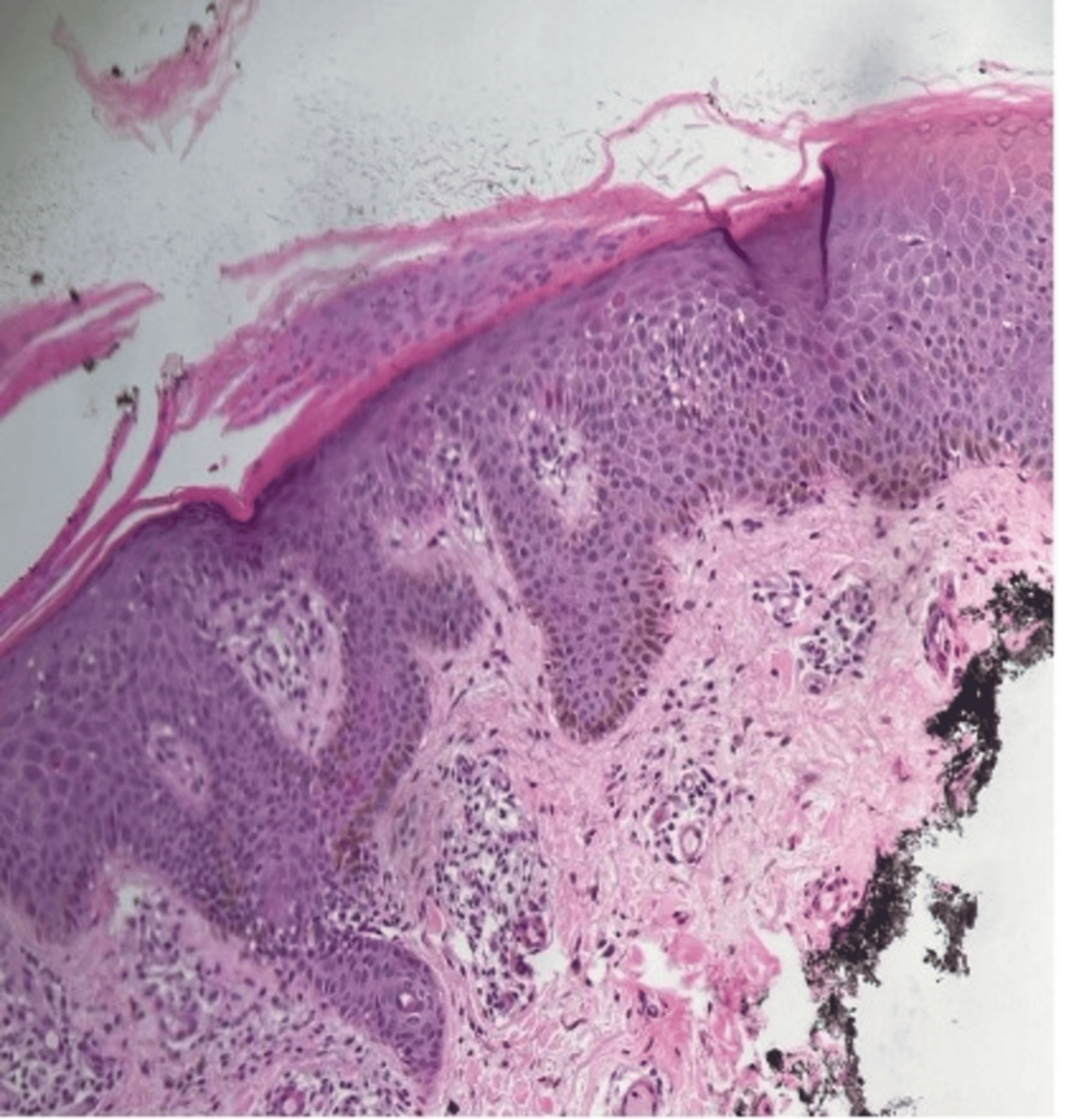
Histology sections show parakeratosis and regular acanthosis, associated with alternating zones of hypogranulosis and hypergranulosis, as well as focal lymphocytic exocytosis within the epidermis. The papillary dermis exhibits vascular dilation with perivascular lymphohistiocytic infiltration

Following her presentation, a skin biopsy was obtained, and it was consistent with psoriasis (Figure 4). The differential diagnosis included palmoplantar eczema, hyperkeratotic tinea pedis, palmoplantar keratoderma, and a drug-induced psoriasiform eruption. Palmoplantar eczema was considered; however, the presence of sharply demarcated plaques with silvery scale, classical extensor involvement, and characteristic nail findings (pitting, oil spots) favored psoriasis. Dermatophytosis was deemed unlikely due to the bilateral symmetrical distribution and absence of active borders; moreover, histopathology did not demonstrate fungal elements. Palmoplantar keratoderma was excluded based on the inflammatory infiltrate and associated extra-palmoplantar lesions. A psoriasiform drug eruption was considered; however, the histopathological findings showing regular acanthosis, parakeratosis with hypogranulosis, and vascular dilation in the papillary dermis were consistent with plaque psoriasis rather than a nonspecific psoriasiform dermatitis.

The patient was managed with topical betamethasone-calcipotriol cream, salicylic acid, and tacrolimus 0.1% twice daily for six weeks. She showed significant improvement with topical therapy and continued the treatment for approximately four months. During her follow-up six months later, her lesions had completely resolved two weeks after stopping letrozole. However, nail onychorrhexis persisted due to slow nail growth. We expect full recovery of nail findings within 9-12 months, once the nail plate has fully regrown.

Case 2

A 48-year-old female with dyslipidemia and left breast invasive ductal carcinoma (luminal A/B cT4bN0-1Mx, estrogen- and progesterone-receptor positive) completed neoadjuvant chemotherapy consisting of doxorubicin, cyclophosphamide, and paclitaxel (12 doses), followed by a left mastectomy with axillary dissection and eight sessions of radiation therapy.

Regarding hormonal therapy, she was on tamoxifen and abemaciclib. On November 26, she was started on letrozole. Two weeks later, she developed a skin eruption on the cubital fossa, inframammary region, and upper thighs. She presented to the dermatology clinic on January 6, 2025, where she reported no personal or family history of psoriasis and no prior or current joint complaints. She had undergone an appendectomy in January 2023 and was not on any medication for dyslipidemia.

**Figure 5:**
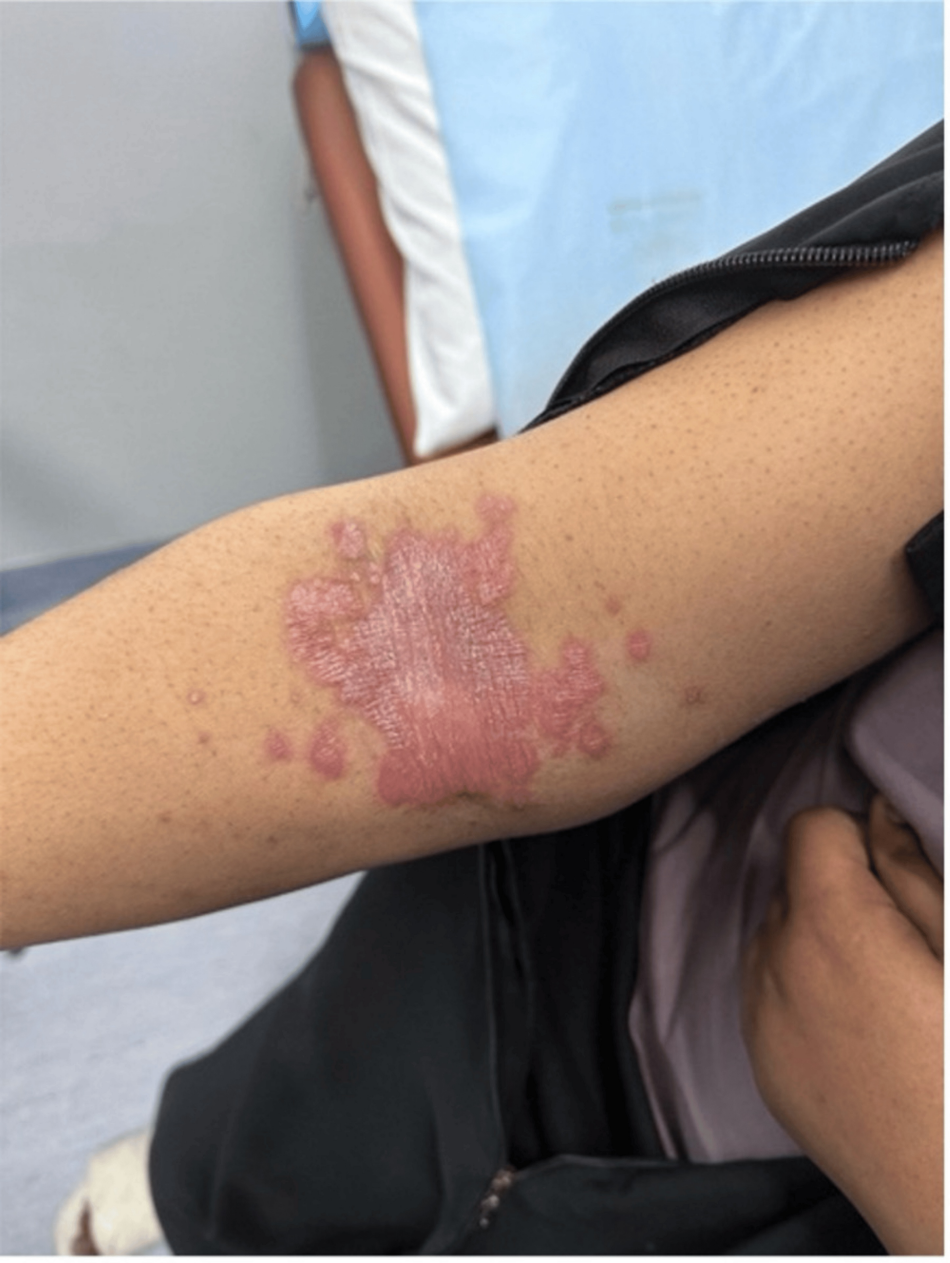
The right cubital fossa

**Figure 6:**
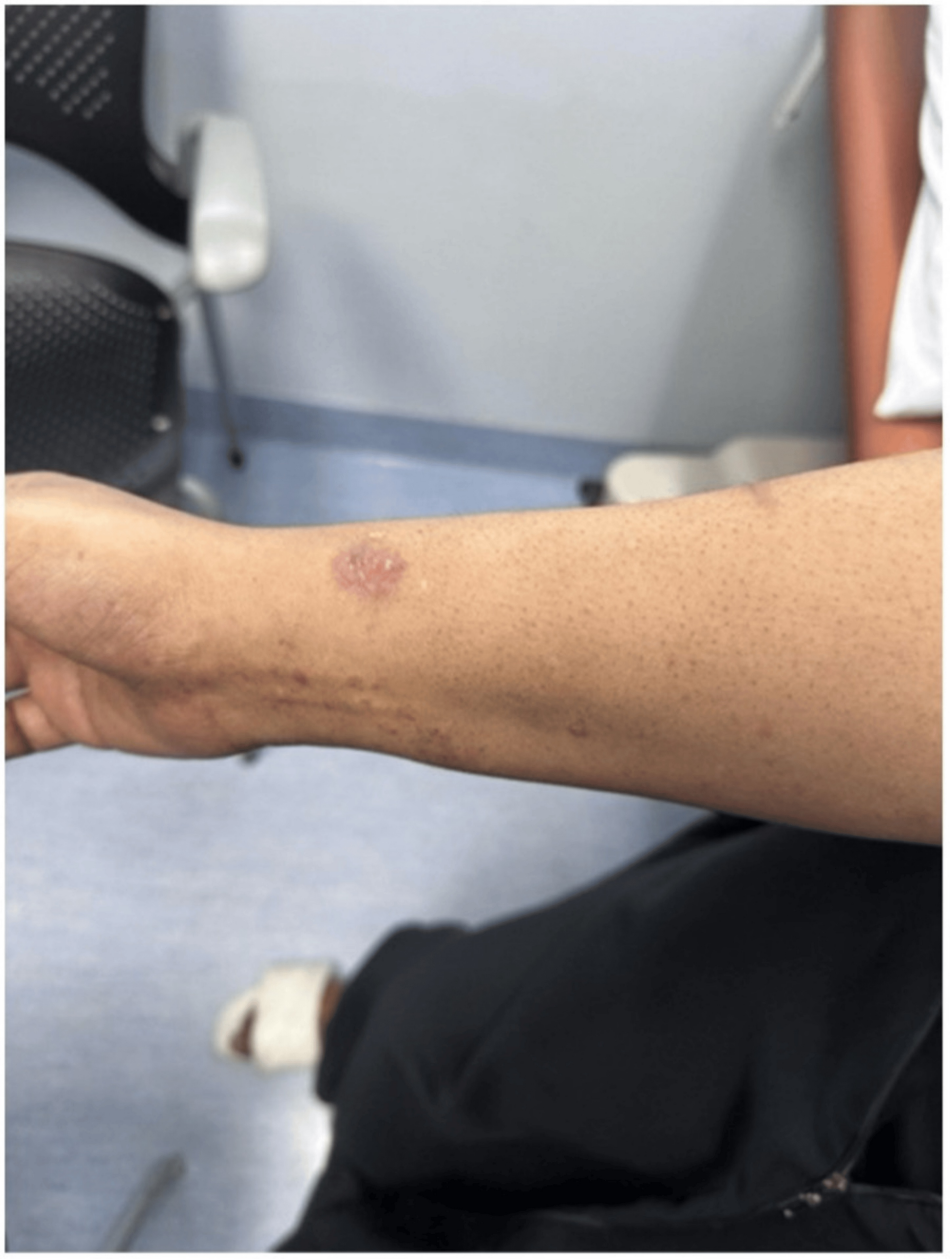
Right medial forearm

Upon physical examination, she had bilateral, well-defined erythematous plaques with silvery scales over the cubital fossa and medial forearm (Figures 5, 6), along with less scaly lesions in the inframammary area and inguinal folds and scattered erythematous papules over the thighs and abdomen. She also had brown pigmentation across all nails, onycholysis of the big toe, and subungual hyperkeratosis. The patient noted that these nail changes had been present since the initiation of neoadjuvant chemotherapy.

**Figure 7:**
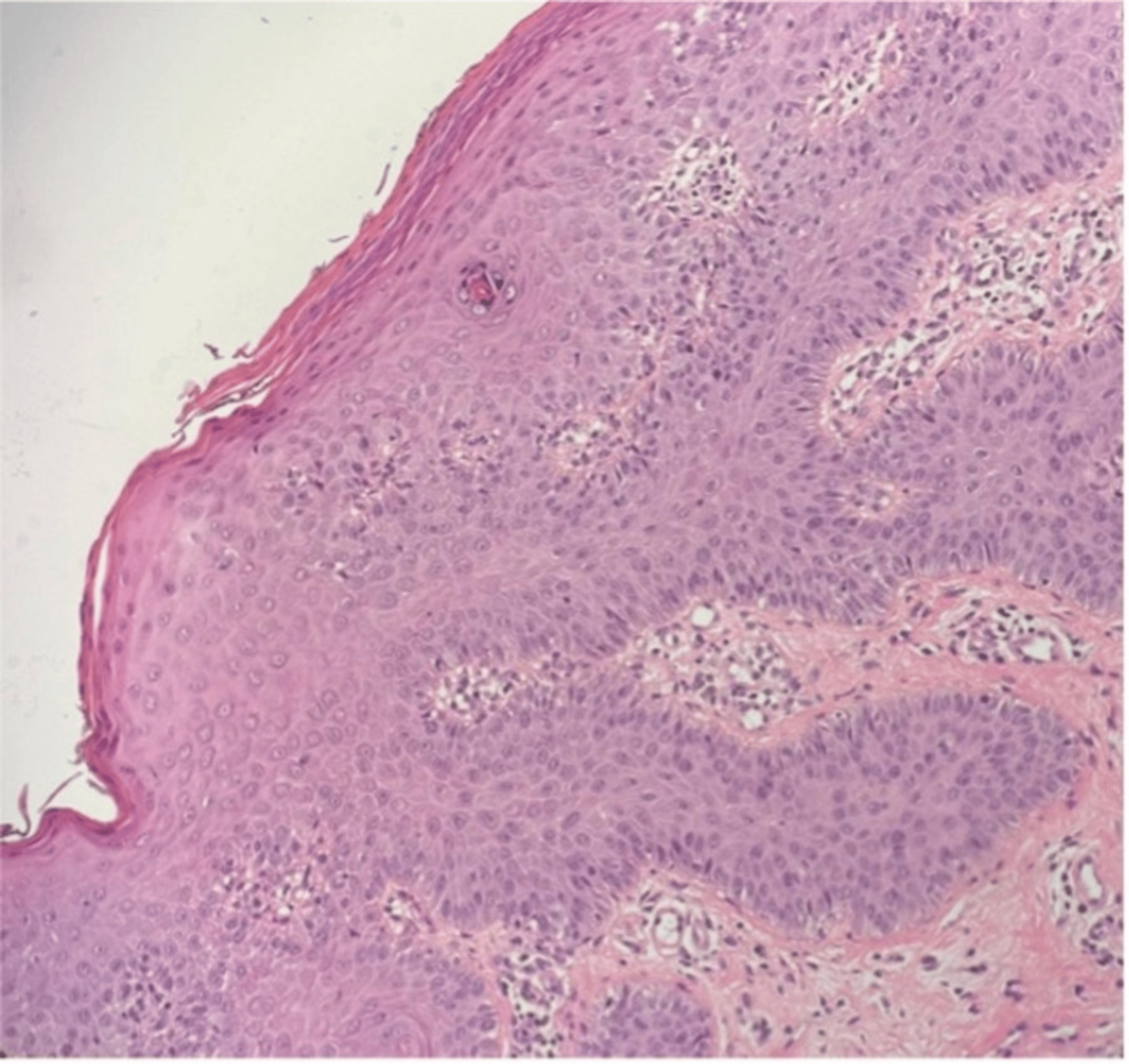
: Histology sections show acanthosis with elongated rete ridges and alternating zones of hypergranulosis in the epidermis. Areas of parakeratosis in the stratum corneum were observed, along with a perivascular, predominantly lymphocytic infiltrate in the upper and middle portions of the dermis. Additionally, mildly dilated and tortuous vessels were present in the dermal papillae. Thinning of the suprapapillary plates, widened vessels, and pigment incontinence in the superficial dermis were also noted

The differential diagnosis included inverse psoriasis, intertriginous candidiasis, seborrheic dermatitis, pityriasis rosea, and a drug-related psoriasiform eruption. Intertriginous candidiasis was considered due to involvement of the inframammary and inguinal folds; however, the absence of satellite pustules favored psoriasis. Seborrheic dermatitis was less likely given the extent of involvement and supportive histological findings. Pityriasis rosea was excluded based on the absence of a herald patch and the chronicity of lesions. Although nail changes were present, these preceded letrozole initiation and were attributed to prior chemotherapy rather than active psoriatic nail disease. Histopathological examination confirmed features consistent with psoriasis (Figure 7).

The patient was prescribed topical betamethasone-calcipotriol gel for intertriginous areas and cream for the abdomen and thighs. Two weeks later, she showed marked improvement. Furthermore, her treatment was switched to tamoxifen instead of letrozole, leading to complete clearance of the skin lesions.

A summary of the clinical features, histopathology, treatment, and outcomes of both cases is provided in Table 1, allowing for easy comparison of key characteristics, latency, and responses to therapy. 

**Table 1:** Summary of clinical features, histopathology, treatment, and outcomes of two patients with letrozole-induced plaque psoriasis This table summarizes patient demographics, comorbidities, breast cancer history, hormonal therapy, time to onset of psoriasis, clinical and histopathological features, initial treatment, and outcome. HTN: hypertension; chemo: chemotherapy; N/A: not applicable.

Feature	Case 1	Case 2
Age / Sex	69-year-old female	48-year-old female
Comorbidities	Hypertension	Dyslipidemia
Breast Cancer History	Relapsed ductal carcinoma in situ, right mastectomy; prior triple-negative breast cancer treated with lumpectomy, chemo, and radiotherapy	Invasive ductal carcinoma (luminal A/B, left breast), neoadjuvant chemo, mastectomy, radiation
Hormone Therapy	Letrozole 2.5 mg daily	Letrozole (after tamoxifen + abemaciclib)
Time to Onset of Psoriasis	6 months after Letrozole initiation	2 weeks after Letrozole initiation
Personal / Family History of Psoriasis	None	None
Clinical Features	Bilateral pink plaques with silvery scales over palms, soles, elbows, knees; nail changes: pitting, oil spots, onychorrhexis (all nails)	Bilateral erythematous plaques with silvery scales over cubital fossa, medial forearm; less scaly lesions in inframammary area & inguinal folds; nail changes: brown pigmentation, onycholysis of big toe, subungual hyperkeratosis (present since chemo)
Histology	Parakeratosis, regular acanthosis, alternating hypogranulosis/hypergranulosis, focal lymphocytic exocytosis, vascular dilation with perivascular lymphohistiocytic infiltrate	Acanthosis with elongated rete ridges, alternating hypergranulosis, parakeratosis, perivascular lymphocytic infiltrate, dilated tortuous dermal papillae vessels, thinning of suprapapillary plates, pigment incontinence
Initial Treatment	Topical betamethasone-calcipotriol cream, salicylic acid, tacrolimus 0.1% for six weeks (continued 4 months)	Topical betamethasone-calcipotriol gel/cream for affected areas
Outcome	Complete clearance of lesions 2 weeks after Letrozole cessation; nail onychorrhexis persists, expected full recovery in 9–12 months	Marked improvement in 2 weeks; complete clearance after switching from Letrozole to tamoxifen
Latency / Trigger	Likely drug-induced due to Letrozole	Likely drug-induced due to Letrozole

## Discussion

Psoriasis is a chronic immune-mediated inflammatory disorder influenced by genetic, environmental, and immunological factors. Drug-induced psoriasis is a recognized entity and may present either as new-onset disease or as an exacerbation of pre-existing psoriasis. Several medications have been implicated, including lithium, interferons (INFs), anti-PD-1 antibodies, and beta-blockers. Systemic corticosteroids have also been associated with psoriasis flares, particularly during rapid tapering.

Letrozole, a nonsteroidal aromatase inhibitor, is widely prescribed as first- and second-line endocrine therapy for hormone receptor-positive breast cancer due to its cost-effectiveness and favorable tolerability profile [2]. Common adverse effects include hot flushes, back pain, headache, nausea, dyspnea, peripheral edema, and fatigue [4]. Dermatological adverse events are uncommon and generally nonspecific, such as pruritus or rash. More severe cutaneous reactions have been reported rarely, including erythema multiforme, toxic epidermal necrolysis, lichenoid drug eruption, and other inflammatory dermatoses [5-15]. However, plaque-type psoriasis associated with letrozole appears to be exceedingly rare.

Alternative explanations

When evaluating these cases, alternative explanations must be considered to strengthen causal inference. One possibility is a paraneoplastic phenomenon. Although paraneoplastic dermatoses are well described in association with malignancy, psoriasis is not classically recognized as a paraneoplastic manifestation of breast carcinoma [2]. Furthermore, in both patients, the malignancy had been surgically treated prior to the eruption, and there was no clinical evidence of tumor progression at the time of psoriasis onset. The temporal relationship between letrozole initiation and subsequent resolution after drug discontinuation argues against a paraneoplastic mechanism.

Another consideration is the spontaneous development of idiopathic psoriasis coinciding with treatment cessation. Psoriasis may arise de novo in adulthood without family history [2]. However, the absence of prior personal or familial psoriasis, the relatively close temporal association with letrozole initiation (six months in Case 1 and two weeks in Case 2), and complete clearance following withdrawal collectively make spontaneous idiopathic psoriasis less likely. While spontaneous remission of psoriasis can occur, it is typically unpredictable and not consistently linked to medication discontinuation, as observed in both cases.

Taken together, although definitive causality cannot be established in a case series, the chronological association, histopathological confirmation, and reproducible improvement after cessation support a probable drug-induced phenomenon.

Potential mechanisms and the role of estrogen in cutaneous immunity

The mechanism by which letrozole may induce psoriasis is not fully elucidated; however, estrogen depletion provides a biologically plausible explanation. Estrogen exerts significant immunomodulatory effects and plays an important role in maintaining cutaneous immune homeostasis [16,17]. It influences keratinocyte proliferation, skin barrier integrity, and cytokine production [17]. Estrogen has been shown to modulate T-cell differentiation and cytokine balance, including pathways involved in Th1- and Th17-mediated immune responses, which are central to psoriasis pathogenesis [17,18].

Aromatase inhibitors such as letrozole profoundly reduce circulating estrogen levels by blocking peripheral conversion of androgens to estrogens [4]. This abrupt hormonal alteration may shift the immune balance toward a pro-inflammatory state, potentially enhancing cytokine signaling pathways implicated in psoriasis [18]. In susceptible individuals, this immune dysregulation may trigger the development of psoriatic lesions.

The variation in latency observed between our two cases may reflect differences in individual immune responsiveness, cumulative hormonal changes, or subclinical predisposition. Nevertheless, both cases shared a similar plaque-type phenotype and favorable response after letrozole withdrawal, supporting a common triggering mechanism.

Clinical implications

With the increasing use of aromatase inhibitors in breast cancer management, clinicians should remain aware of the possibility of rare inflammatory dermatologic reactions, including psoriasis. Early recognition is important, as discontinuation or substitution of the offending agent may result in complete resolution without the need for systemic psoriasis therapy.

Further pharmacovigilance data and larger studies are needed to better characterize this association and to determine whether specific patient-related or regional factors may predispose individuals to this uncommon adverse effect. The occurrence of two cases within a single institution may raise the question of potential racial or regional susceptibility. Psoriasis prevalence and clinical expression are known to vary among different ethnic groups and geographic regions [2]. It is possible that genetic background, environmental exposures, or regional prescribing patterns contributed to the identification of these cases at our center. However, given the limited number of cases, it is not possible to draw definitive conclusions regarding a specific racial or regional predisposition. Further studies involving larger and more diverse populations would be required to clarify this observation.

We report two cases of new-onset plaque psoriasis in patients with no identifiable risk factors, including personal or family history of psoriasis. Genetic predisposition, such as HLA typing, was not assessed. The latency between letrozole initiation and psoriasis onset differed between the cases, occurring six months after starting therapy in Case 1 and just two weeks in Case 2; despite this, both patients exhibited a consistent plaque-type psoriasis phenotype. Importantly, discontinuation of letrozole led to complete resolution of cutaneous lesions in both patients. In Case 1, follow-up at six months confirmed sustained clearance, with nail changes expected to fully recover within 9-12 months. In Case 2, dermatologic improvement was observed within two weeks after switching to tamoxifen, and follow-up monitoring was planned every 2-3 months to detect any potential recurrence.

Taken together, these observations support letrozole as the most likely trigger for the psoriatic eruptions and highlight the need for ongoing dermatologic surveillance after cessation or substitution of the offending agent.

## Conclusions

Drug-induced psoriasis has been associated with various medications. While letrozole is linked to multiple cutaneous manifestations, no previous reports have documented psoriasis as an adverse effect. Thus, we report psoriasis as a potential cutaneous reaction to letrozole. Clinicians should maintain a high index of suspicion when skin eruptions appear shortly after starting a new drug. Early recognition and discontinuation of the offending medication, if possible, are crucial for management.
